# Serum midkine as a biomarker for malignancy, prognosis, and chemosensitivity in head and neck squamous cell carcinoma

**DOI:** 10.1002/cam4.600

**Published:** 2016-01-22

**Authors:** Taku Yamashita, Hideaki Shimada, Shingo Tanaka, Koji Araki, Masayuki Tomifuji, Daisuke Mizokami, Nobuaki Tanaka, Daisuke Kamide, Yoshihiro Miyagawa, Hiroshi Suzuki, Yuya Tanaka, Akihiro Shiotani

**Affiliations:** ^1^ Department of Otorhinolaryngology‐Head and Neck SurgeryNational Defense Medical CollegeTokorozawaSaitama359‐8513Japan; ^2^ Department of SurgerySchool of MedicineToho University Omori Medical CenterTokyo143‐8541Japan

**Keywords:** Biomarker, enzyme‐linked immunosorbent assay, immunohistochemistry, induction chemotherapy, tumor marker

## Abstract

Improved therapies for individuals with head and neck squamous cell carcinoma (HNSCC) may be developed by identification of appropriate biomarkers. The aim of this study was to evaluate the usefulness of serum midkine measurement as a biomarker for HNSCC. Pretreatment serum midkine concentrations were measured in 103 patients with HNSCC and 116 control individuals by enzyme‐linked immunosorbent assay. Midkine expression in tumor tissues from 33 patients with HNSCC who underwent definitive surgical resection without preoperative treatment was examined by immunohistochemistry. The cut‐off serum midkine concentrations for predicting the presence of head and neck malignancy and chemosensitivity to induction chemotherapy, as determined using receiver operating characteristic curves, were 482 and 626 pg/mL, respectively. Spearman bivariate correlations showed positive correlations between serum midkine levels and immunohistochemistry staining score (*r* = 0.612, *P *<* *0.001). The sensitivity, specificity, positive predictive value, negative predictive value, and accuracy of serum midkine concentration for detection of HNSCC were 57.3, 85.3, 77.6, 69.2, and 72.1%, respectively. However, for predicting the response to induction chemotherapy, the values were 84.6, 60.9, 71.0, 77.8, and 73.5%, respectively. Serum midkine concentration was identified as an independent prognostic factor by multivariate analysis, using Cox's proportional hazards model (*P *=* *0.027). Overexpression of serum midkine yielded a relative risk of death of 3.77, with 95% confidence limits ranging from 1.15 to 17.0. Serum midkine levels in patients with HNSCC were associated with malignancy, chemosensitivity, and prognosis. Serum midkine may be a useful, minimally invasive biomarker for early detection, therapeutic decision‐making, and predicting prognosis.

## Introduction

Head and neck squamous cell carcinoma (HNSCC) is the seventh most frequent cancer in the world, with over 680,000 patients diagnosed annually 1. Despite recent progress in the diagnosis and multidisciplinary treatment of HNSCC, the 5‐year survival rate has remained between 50 and 60% worldwide over the past two decades 2. However, recent studies have reported the early detection of head and neck cancers owing to the progression of diagnostic technologies, such as positron‐emission tomography (PET), high‐resolution electronic endoscopy, and optical imaging technologies (e.g., narrow band imaging [NBI] and i‐scan) 3, 4, 5, 6. Early detection is the key to improved prognosis in patients with HNSCC. In addition, transoral surgery for early local stage pharyngeal and laryngeal cancer, such as transoral robotic surgery (TORS) 7, 8, 9, transoral laser microsurgery (TLM) 10, 11, 12, and transoral videolaryngoscopic surgery (TOVS) 13, 14, 15, has become popular worldwide as a minimally invasive surgery, allowing for the preservation of function. Thus, early detection of HNSCC provides considerable benefits to patients, including improved prognoses and functional preservation in deglutition, phonation, and articulation by transoral resection of local tumors. However, the use of PET or high‐resolution electronic endoscopy for all patients as a cancer screening tool is not practical due to the invasiveness, cost, and burden to medical staff. Therefore, an appropriate blood test to monitor tumor biomarkers for early detection of HNSCC would be ideal. Although some molecules have been reported as serum tumor markers for HNSCC, the sensitivity of these molecules for early detection of HNSCC is insufficient. The development of more sensitive tumor markers for the detection of early stage HNSCC would improve cancer screening and posttreatment follow up.

Midkine (MK), a heparin‐binding growth factor, was first discovered as a highly expressed factor involved in embryonic development;^16^ since its discovery, MK has been reported to be overexpressed in at least 20 different types of cancer 17. Studies have shown that MK or a combined biomarker test including MK outperforms other current serum biomarkers in terms of sensitivity for early detection of various malignant tumors, such as esophageal squamous cell carcinoma 18, 19, hepatocellular carcinoma 20, and colorectal carcinoma 21. In addition, MK promotes many tumor‐specific functions, such as cell growth, tumor cell survival, cell migrations, and carcinogenesis 22, 23. Several studies have demonstrated the importance of MK in tumorigenesis. It has been reported that MK overexpression by transfecting NIH3T3 fibroblasts with an MK expression vector can transform the NIH3T3 fibroblasts into fibrosarcoma cells via an unknown mechanism 24. Furthermore, genetic ablation of MK delayed tumor formation and reduced tumor incidence in a transgenic neuroblastoma model through attenuation of the Notch2 signaling pathway 25. Although the mechanisms by which MK induces tumorigenesis and tumor advancement have not been fully elucidated, several have been proposed. These include induction of cancer cell proliferation, cell survival, anti‐apoptosis, tumorigenesis, and epithelial‐mesenchymal transition (EMT). Several studies have showed that these functions of MK are derived from activation of the mitogen‐activated protein kinase (MAPK) and phosphatidylinositol 3‐kinase (PI3K)/Akt pathways by the MK receptor anaplastic lymphoma kinase (ALK), activation of the extracellular signal‐regulated kinase 1/2 (ERK1/2), and PI3K pathways via protein tyrosine phosphatase *ζ* (PTP*ζ*) (another MK receptor), activation of the Janus tyrosine kinase (JAK)/signal transducer, and activator of transcription (STAT) pathway, as well as activation of Notch signaling 26.

Because MK is a soluble and secreted protein lacking any sort of membrane spanning domain 17, serum MK concentration is expected to increase with respect to the presence of MK‐expressing tumors. Although overexpression of serum MK protein is associated with poor prognosis in some types of cancer 18, 27, the significance of serum MK in HNSCC has not yet been investigated.

Therefore, in this study, we aimed to determine the relevance of serum MK as a simple biomarker for detection of malignancy, determination of prognosis, and prediction of sensitivity to induction chemotherapy (ICT) in patients with HNSCC.

## Patients and Methods

### Patients and controls

One hundred and three patients newly diagnosed with HNSCC between February 2012 and August 2014 at the Department of Otolaryngology‐Head and Neck Surgery, National Defense Medical College of Japan were enrolled in this study. The patients included 82 men (79.6%) and 21 women (20.4%) with a median age of 68 years (range, 36–92 years). The TN classifications of all malignant cases are shown in Table 1. One hundred cases (97.1%) were M0, and three cases (2.9%) were M1. The clinical stages at the time of diagnosis were as follows: two patients with stage 0, 13 patients with stage I, 14 patients with stage II, 15 patients with stage III, and 59 patients with stage IV. Although there are no criteria published by the American Joint Committee on Cancer (AJCC) regarding stage classification for external auditory canal (EAC; *n* = 2) and occult primary (OP; *n* = 4) cases, we defined their stages in this study, using the Pittsburgh classification 28 for T classification of EAC cases and the Seventh International Union Against Cancer (UICC) TNM staging system of oropharyngeal or hypopharyngeal cancer for N and M classifications for OP cases. Subsequently, we decided the stages of the six cases according to the AJCC staging system of oropharyngeal or hypopharyngeal cancer. All patients underwent primary treatment for HNSCC in our department. The median follow‐up duration of censored cases was 20 months.

**Table 1:** TN classification for HNSCC cases

	N0	N1	N2	N3	Total
T0	0	0	1	3	4
Tis	2	0	0	0	2
T1	13	1	1	1	16
T2	15	2	12	1	30
T3	7	2	11	1	21
T4	6	4^a^	18	2^b^	30
Total	43	9	43	8	103

a Including two M1 cases.

b Including one M1 case.

One hundred and sixteen individuals who were healthy volunteers or visited our clinic with benign otolaryngological disease were also included in this study as controls. Individuals who had malignant disease within 5‐years were excluded from control cases. No patient or control case had received blood transfusion, radiotherapy, or chemotherapy during the 12‐month period prior to the study. The control cases included 53 men (45.7%) and 63 women (54.3%), with a median age of 64 years (range, 22–80 years). In this control population, 34 were healthy volunteers (29.3%), 21 had benign tumors of the head and neck region (18.1%), 23 had otolaryngological inflammatory diseases (19.8%), and 38 had other benign diseases, such as Bell's palsy, sudden deafness, and Meniere's disease (32.8%).

### Serum sampling and enzyme‐linked immunosorbent assay for measurement of MK

After obtained written informed consent, the blood of patients and controls was collected and left it at room temperature. Subsequently, samples were centrifuged at 5000 ***g*** for 5 min. Serum was collected and kept frozen at −80°C until the assays were performed. An enzyme‐linked immunosorbent assay (ELISA) kit for human MK (CDYELISA, Cellmid Ltd., Sydney, Australia) was used to detect serum MK concentrations. We determined serum MK levels in the collected samples according to the manufacturer's protocol. The absorbance was measured with a microplate reader (2030 ARVO X; PerkinElmer Inc., Waltham, MA) at a wavelength of 450 nm and analyzed using WorkOut 2.5 software (PerkinElmer Inc., Waltham, MA).

### Immunohistochemistry

For the comparison of MK protein expression in tumor tissues with serum MK concentrations, immunohistochemistry (IHC) was performed using surgical excision specimens. The specimens were fixed with 15% formaldehyde for more than 48 h, and paraffin‐embedded tissue blocks were prepared. IHC was performed using 4 *μ*m‐thick tumor sections made from these paraffin blocks. Heat‐induced antigen retrieval was carried out, using deparaffinized sections with 10 mmol/L citrate buffer (pH 6.0) at 95°C for 15 min. Endogenous peroxidase activity was blocked by incubating the sections in 3% hydrogen peroxide in methanol for 10 min. The primary antibody was an anti‐MK rabbit monoclonal antibody (IgG; clone EP1143Y; 1:200 dilution; Novus Biologicals, Littleton, CO.). The slides were incubated with the primary antibody overnight at 4°C, and the specific reactions were then detected using a VECTASTAIN Elite ABC kit (PK‐6101; Vector Laboratories) and ImmPACT DAB (SK‐4105; Vector Laboratories, Burlingame, CA). Subsequently, the nuclei were counterstained with hematoxylin 3G (#8656; Sakura Finetek, Japan). Negative control sections were incubated with phosphate‐buffered saline (PBS) instead of primary antibodies. Immunoreactivity for MK was quantitatively scored according to the percentage of positive cells and staining intensity using the following criteria^29^. The staining positivity percentage (positively stained tumor cells/all tumor cells) was scored based on four categories: 0 (<5%), 1 (6–35%), 2 (36–70%), and 3 (>71%). The staining intensity as a whole was also scored based on four categories: 0 (none), 1 (weak), 2 (moderate), and 3 (intense). Immunoreactivity was then evaluated according to a combination of the two parameters above, with scores of 0–6 as the sum of the positivity, and intensity scores. The staining was assessed by two physicians who were blinded to the patient's clinical information, including the serum MK levels. We determined the mean value calculated from the evaluation by two physicians as an IHC score for each case.

### SCC antigen and CYFRA21‐1 assay

We also measured SCC antigen and CYFRA21‐1, which are traditional tumor markers for HNSCC covered by the Japanese public health insurance, using simultaneously corrected blood samples for the same HNSCC cases. Serum SCC antigen concentrations were measured using an automatic chemiluminescence immunoassay analyzer (ARCHITECT i2000; Abbott Japan, Tokyo, Japan). Serum CYFRA21‐1 concentrations were evaluated using a fully automated chemiluminescent immunoassay system (Lumipulse Presto; FUJIREBIO Inc., Tokyo, Japan). The cut‐off values of SCC and CYFRA in our hospital are 1.5 and 3.5 ng/mL, respectively.

### Statistical analysis and ethical issues

Mann–Whitney *U* tests, Kruskal–Wallis tests, chi‐square tests for independence, and log‐rank tests were conducted for univariate analysis. Differences or correlations with *P*‐values of <0.05 were considered statistically significant. Statistical analyses were carried out in Microsoft Excel for Windows 2010 (Microsoft, Redmond, WA) with the add‐in Statcel 3 software (OMS, Saitama, Japan). Multivariate logistic regression analysis was performed with JMP statistical package version 10 (SAS Institute, Cary, NC). Cumulative overall survival and recurrence rates in patients with normal or abnormal serum MK were calculated using the Kaplan–Meier method. The determination of cut‐off values of serum MK for prediction of the presence of malignancy and the response to ICT was carried out using receiver operating characteristic (ROC) curves as the value that maximized the Youden's index (sensitivity + specificity −1).

This study was performed in accordance to the Declaration of Helsinki and was approved by the ethical committee of the National Defense Medical College of Japan. All patients and controls provided written informed consent to participate in this study.

## Results

### Serum MK concentrations and clinical features of patients and controls

**Figure 1:**
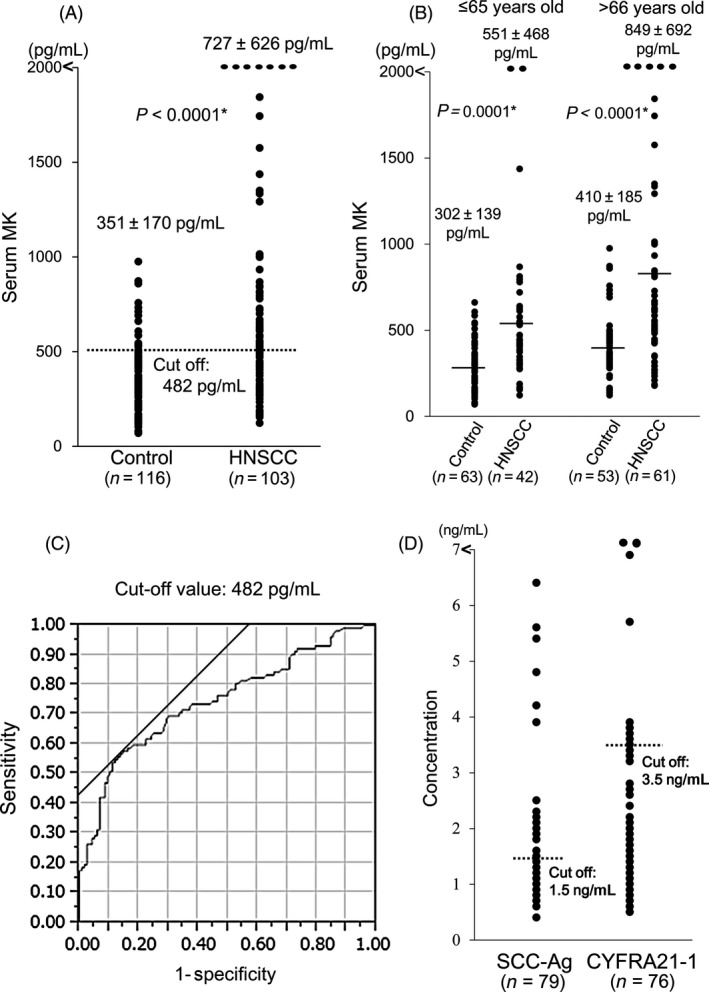
Serum midkine concentrations for control individuals and patients with head and neck squamous cell carcinoma (HNSCC) for all cases (A) and for cases divided into subgroups based on age (≤65 and >65 years) (B) *Mann–Whitney *U* test. The cut‐off serum Midkine (MK) value (482 pg/mL) predicting the presence of head and neck malignancy was established using the receiver operating characteristic curve (C). The distribution of traditional tumor markers for HNSCC, SCC antigen, and CYFRA 21–1(d). The cutoff values of the SCC antigen and CYFRA 21–1 were 1.5 and 3.5 ng/mL, respectively.

The clinical features of all study participants are shown in Tables 1 and 2. In controls, serum MK concentrations were significantly higher in individuals more than 65 years old than in individuals of 65 years old or younger (Table 2). In patients with HNSCC, there were no correlations between serum MK concentration and primary site, T classification, N classification, M classification, or clinical stage. However, age, gender, and Karnofsky performance status score were significant factors influenced by serum MK levels (Table 3). Midkine concentrations were significantly different between patients with HNSCC and controls (*P *<* *0.0001; Fig. 1A). Moreover, in both age groups, serum MK concentrations were significantly different between patients with HNSCC and controls (*P *=* *0.0001 in the ≤65 years old group, and *P *<* *0.0001 in the >65 years old group; Fig. 1B). From our data, the cut‐off serum MK concentration predicting the presence of head and neck malignancy was 482 pg/mL (Fig. 1C). Sensitivity, specificity, positive predictive value, negative predictive value, and accuracy of serum MK for patients with HNSCC were 57.3, 85.3, 77.6, 69.2, and 72.1%, respectively, using the cut‐off value of 482 pg/mL. The sensitivity of SCC antigen and CYFRA21‐1, which were measured for patients with HNSCC using simultaneously collected blood samples, were 35.4 and 13.1%, respectively (Fig. 1D). No correlation was observed between serum MK levels and SCC‐Ag concentrations (Spearman correlation coefficient: *r* = 0.143, *P *=* *0.214), while a weak correlation was observed between serum MK levels and CYFRA21‐1 concentrations (Spearman correlation coefficient: *r* = 0.317, *P *=* *0.006).

**Table 2:** Clinical variables in 116 control cases

Variables		No. of patients	serum MK (pg/mL)	*P* value
Gender	Male	53	371 ± 196	0.483^a^
	Female	63	334 ± 114	
Age	≤65	63	302 ± 139	0.001^a^
	>65	53	410 ± 185	
Diseases	Healthy individuals	34	338 ± 171	0.732^b^
	Benign tumors	21	394 ± 207	
	Inflammatory diseases	23	349 ± 112	
	Other diseases	38	340 ± 178	

a Mann–Whitney *U* test.

b Kruskal–Wallis test.

**Table 3:** Clinical variables in 103 patients with HNSCC

Variables		No. of patients	Serum MK(pg/mL)	*P* value
Gender	Male	82	758 ± 614	0.030^a^
	Female	21	607 ± 671	
Age	≤65	42	551 ± 468	0.002^a^
	>65	61	849 ± 692	
Karnofsky scale	≥80	86	660 ± 546	0.032^a^
	≤70	17	1065 ± 875	
Primary site	Oral	10	833 ± 738	0.669^b^
	Oropharynx	17	952 ± 881	
	Hypopharynx	38	730 ± 587	
	Larynx	25	637 ± 532	
	The others	13	517 ± 330	
T classification	T0‐2	52	654 ± 584	0.129^a^
	T3‐4	51	802 ± 663	
N classification	N0‐1	52	767 ± 746	0.753^a^
	N2‐3	51	687 ± 478	
M classification	M0	100	721 ± 627	0.456^a^
	M1	3	951 ± 651	
Clinical Stage	0–II	29	728 ± 732	0.350^a^
	III–IV	74	727 ± 585	

a Mann–Whitney *U* test.

b Kruskal–Wallis test.

### Correlations between serum MK levels and immunohistochemical analysis of MK expression in tumor tissues

**Figure 2:**
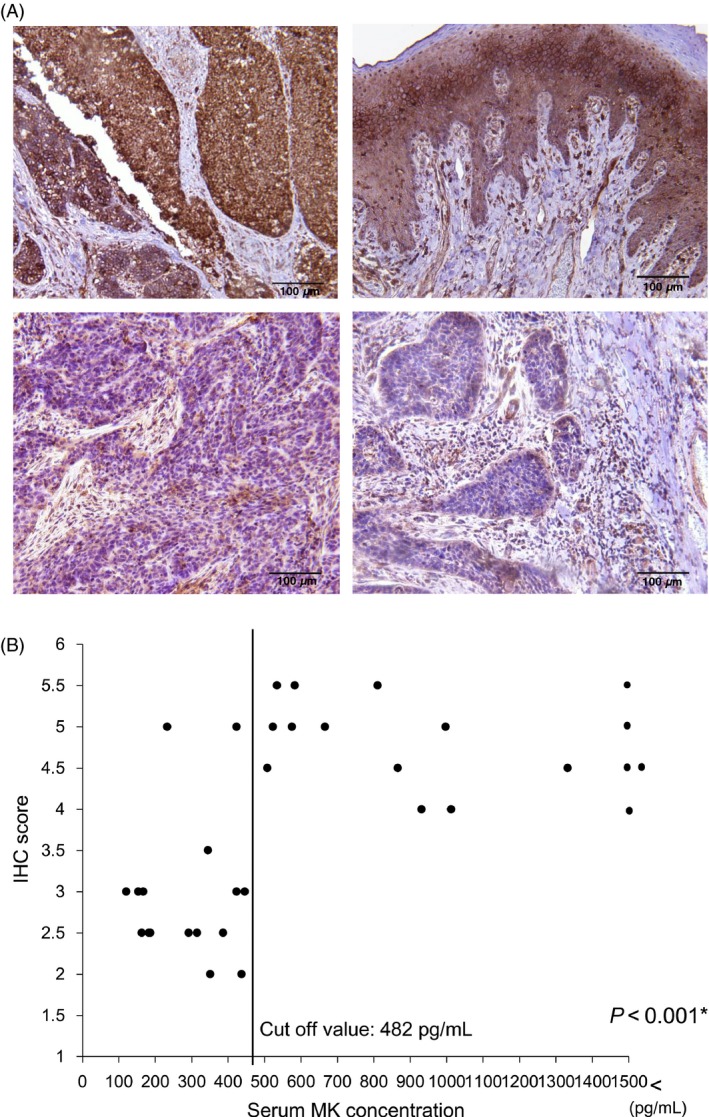
(A) Representative IHC results of tumor specimens with high or low expression of Midkine (MK). The mean IHC scores were 5.5, 5.5, 3.0, and 2.5 in the upper left, upper right, lower left, and lower right specimens, respectively. (B) Relationship between serum MK concentration and IHC scores in 33 surgically excised tumor specimens (Spearman's correlation coefficient: *r* = 0.612, *P *<* *0.001).

Next, we quantified the expression of MK using IHC analysis in 33 samples collected during surgical resection without preoperative treatment. Spearman bivariate correlations showed positive correlations between serum MK levels and IHC staining score (Fig. 2; Spearman correlation coefficient: *r* = 0.612, *P *<* *0.001).

### Serum MK as a predictor of chemosensitivity in HNSCC cases

**Figure 3:**
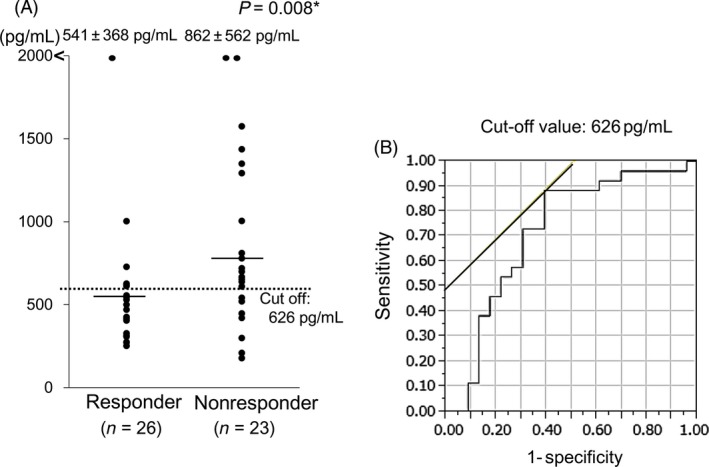
The distribution of serum midkine concentrations of responders and nonresponders for induction chemotherapy in patients with HNSCC (*n* = 49) who underwent induction chemotherapy (A) *Mann–Whitney *U* test. The cut‐off serum Midkine (MK) value (626 pg/mL) predicting the response to induction chemotherapy was established based on the receiver operating characteristic curve (B).

ICT was conducted in 49 out of 103 cases of HNSCC. The used regimen of ICT was TPF (Docetaxel: 60 mg/m^2^ day 1 + Cisplatin: 60 mg/m^2^ day 1 + 5‐fluorouracil: 700 mg/m^2^ day 1–4). The response was estimated using revised Response Evaluation Criteria in Solid Tumors (RECIST) guideline version 1.1 30. The response (complete or partial response) rate was 53.1% (26/49 cases). Serum MK levels were significantly different between responders and nonresponders to ICT (*P *=* *0.008; Fig. 3A). Based on these data, the cut‐off serum MK concentration predicting the response to ICT was 626 pg/mL (Fig. 3B). Sensitivity, specificity, positive predictive value, negative predictive value, and accuracy of serum MK levels for predicting for the response in ICT were 84.6, 60.9, 71.0, 77.8, and 73.5%, respectively.

### Serum MK as a predictor of recurrence and survival in HNSCC cases

**Figure 4:**
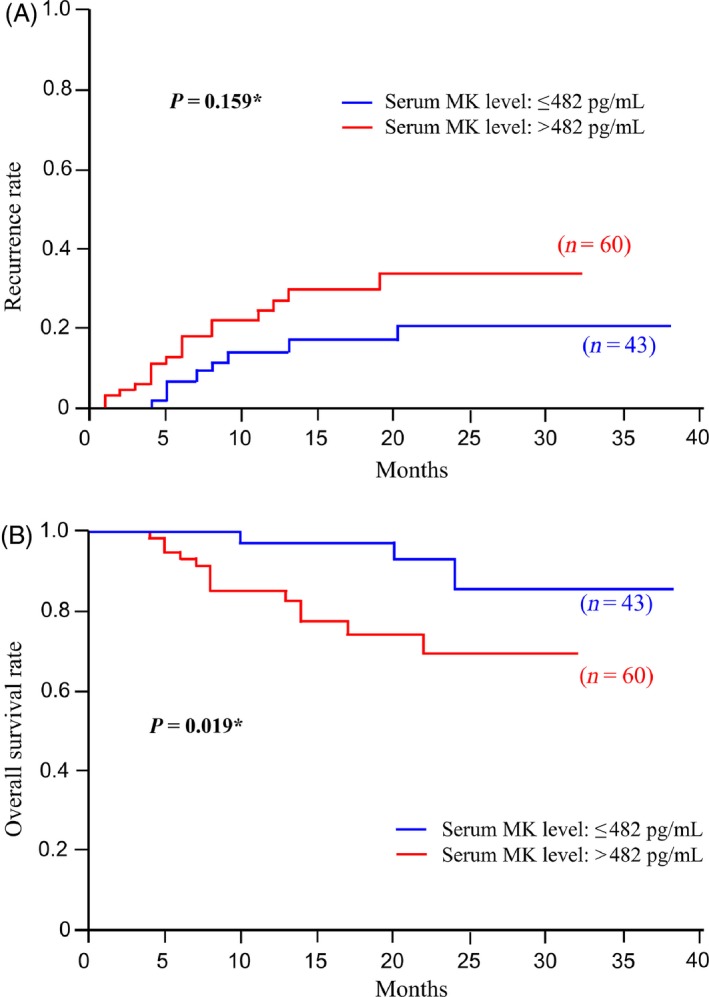
(A) Kaplan–Meier curve of the correlation between recurrence and Midkine (MK) levels (<482 or ≥482 pg/mL). (B) Kaplan–Meier curve of the correlations between overall survival and MK levels (<482 or ≥482 pg/mL). *Log‐rank test.

The correlations of recurrence (Fig. 4A) and survival (Fig. 4B) with MK levels were investigating using the Kaplan–Meier method. Although serum MK levels did not predict recurrence after definitive therapy (*P *=* *0.159), there was significant correlation between serum MK concentration and overall survival. The 2‐year survival rates of patients in the serum MK groups (<482 and ≥482 pg/mL) were 86.2 and 69.7, respectively (*P *=* *0.019).

### Univariate and multivariate analyses for survival in patients with HNSCC

Next, we used univariate analysis to determine the prognostic value of serum MK concentrations and other clinical factors (Table 4). When serum MK concentration, clinical stage, gender, and age were assessed by multivariate analysis using Cox's proportional hazards model, serum MK was identified as an independent prognostic factor (Table 5; *P *=* *0.027). A value of 482 pg/mL or more for serum MK yielded a relative risk of death of 3.77, with 95% confidence limits ranging from 1.15 to 17.0.

**Table 4:** Univariate analysis of patients with HNSCC (*n* = 103)

Variables		No. of patients	2‐year survival rate	*P* value^a^
Gender	Male	82	75.2	0.350
	Female	21	86.4	
Age	≤65	42	74.8	0.891
	>65	61	79.3	
Karnofsky scale	≥80	86	84.7	0.126
	≤70	17	43.3	
Primary site	Oral	10	57.9	0.357
	Oropharynx	17	74.9	
	Hypopharynx	38	71.7	
	Larynx	25	93.8	
	The others	13	81.5	
Clinical stage	0–II	29	88.5	0.065
	III–IV	74	73.5	
Midkine	<482 pg/mL	43	86.2	0.019
	≥482 pg/mL	60	69.7	

a Log‐rank test.

**Table 5:** Multivariate analysis of the survival of HNSCC patients (*n* = 103)

Variables	Hazard ratio	95% CI	*P* value
Midkine	3.77	1.152–17.00	0.027
≥482/<482 (pg/mL)			
Clinical stage	2.61	0.720–16.74	0.158
III, IV/0–II			
Gender	1.76	0.482–11.298	0.429
Male/female			
Age	1.24	0.426–3.438	0.687
≤65/>65 (y/o)			

HNSCC, head and neck squamous cell carcinoma; CI, confidence interval; y/o, years old.

## Discussion

In this study, we aimed to evaluate the applicability of serum MK as a marker for HNSCC. Our data demonstrated that serum MK concentrations were significantly correlated with malignancy, prognosis, and chemosensitivity, consistent with a report using IHC for MK levels for prognosis in patients with HNSCC 31. Moreover, while a previous study examined the usefulness of serum MK concentrations for prognosis in oral squamous cell carcinoma 32, this study is the first report showing the usefulness of circulating serum MK for prediction of prognosis and chemosensitivity for HNSCC primary tumors located at various sites, providing a simple, rapid test that may have benefits clinically.

In this study, with a cut‐off value of 482 pg/mL for predicting the presence of malignancy in HNSCC, the sensitivity and specificity were 57.3 and 85.3%. Although the detection of malignancy was satisfactory, the specificity was somewhat low. In order to achieve 95.0% specificity, the cut‐off value needed to be 660 pg/mL, and the sensitivity was reduced to 32.0%. While overexpression of MK is associated with various malignant neoplasms, normal circulating MK is observed in peripheral blood at a healthy background level 33, 34. In addition, a previous report showed that MK could be elevated in several diseases. In the present study, the control individuals enrolled in our study had a relatively high average age (59.0 years), with 45.7% of control over 65 years of age; this older age may be associated with the presence of undetected underlying chronic diseases, which may elevate MK concentrations. Consistent with this, MK levels in controls older than 65 years of age were significantly higher than those in control 65 years of age or younger. Similar elevation of serum MK levels with increasing age has been observed previously in healthy controls 32. Thus, this may explain the low specificity of MK overexpression for malignancy. Additional studies are required to examine the possibility of different cut‐off values according to age.

In this study, we observed significant correlations between serum MK and MK immunostaining in tumor tissues (Fig. 2B). When we defined 3.5 or more in IHC staining score as immunohistochemically positive, a significant association between serum MK and MK immunoreactivity was observed (Table 6; *P *<* *0.001). This result suggested that serum MK levels were an appropriate measure representing MK protein expression in tumor tissues of patients with HNSCC.

**Table 6:** Associasion between serum Midkine (MK) levels and immunoreactivity by immunohistochemistry (IHC)

	Serum MK (pg/mL)	
IHC positive	17	3
IHC negative	0	13

*P *<* *0.001

Interestingly, no correlation was observed between serum MK concentration and clinical stage in the present study. This finding suggested that overexpression of serum MK could be observed even at a relatively early stage in patients with HNSCC. In fact, overexpression of serum MK (>482 pg/mL) was observed in 44.8% of patients with stage 0–II cancers. Similar tendencies have been reported in various other cancer types, including esophageal squamous cell carcinoma, oral squamous cell carcinoma, gastric cancer, and lung cancer 18, 19, 32, 35, 36. In esophageal squamous cell carcinoma, serum MK has been reported to show higher positive rate than other tumor markers, including SCC‐Ag, CYFRA 21‐1, and VEGF. Moreover, high positive rates are observed, even in early stage cases 35. This observation may be explained by the roles of MK in carcinogenesis 24, angiogenesis 37, tumor growth 22, 23, tumor migration 22, and anti‐apoptotic signaling 38. The expression of SCC antigen, a traditional tumor marker for head and neck cancer, has been reported to be associated with tumor burden and therefore difficult to use for detection of early stage cancer 18, 39. In this study, the sensitivity of SCC antigen for stage 0–II HNSCC cases was only 23.5%, and that of CYFRA 21‐1, another traditional tumor marker for HNSCC and potential early detection marker 40, was only 11.8%. Although the analysis was made only in a small number of cases, the results of this study suggested that serum MK was a more useful tumor marker in terms of early detection for HNSCC than SCC antigen or CYFRA 21‐1. The measurement of serum MK in addition to these traditional tumor markers would be of great value for early detection of malignancy or recurrence of HNSCC.

One of the roles of ICT in HNSCC is chemoselection. Two phase III trials showed that ICT contributes to laryngeal preservation without deteriorating survival in patients with locally advanced resectable HNSCC 41, 42. However, if it were possible to predict response to ICT, nonresponders would greatly benefit from not having to undergo ICT before surgery. In the present study, serum MK levels were associated with chemosensitivity to ICT in patients with HNSCC. With a cut‐off value of 626 pg/mL, the positive predictive value, negative predictive value, and accuracy of predicting the response to ICT were 71.0, 77.8, and 73.5%, respectively. Although the mechanisms mediating this observation is unclear, several proposals have been made. In one study, MK was shown to have cytoprotective effects toward damaging insults by cisplatin, at least in part through enhancement of the expression of Bcl‐2 in Wilms’ tumor 43. Additionally, truncated MK lacking exon 3 has been shown to suppress apoptosis and cell death induced by anticancer agents including cisplatin and paclitaxel through cell‐protective functions 44. Additionally, Kang and colleagues identified over 250 genes differently expressed in 5‐fluorouracil‐, cisplatin‐, and doxorubicin‐resistant gastric cancer cell lines; among them, MK was commonly overexpressed in all drug‐resistant cell lines 45, suggesting that MK is a strong factor contributing to resistance to various chemotherapies.

Several studies have shown that serum or plasma MK concentration is a prognostic marker in various cancer types, including esophageal cancer, oral cancer, neuroblastoma, and hepatocellular carcinoma 18, 27, 32, 46. In this study, we showed that serum MK level was an independent factor associated with survival in patients with HNSCC. Because MK has been reported to be involved in various cancer‐related functions 22, 23, 24, 37, 47, 48, it is easy to understand how MK overexpression may enhance the degree of malignancy and how MK expression could be associated with poor prognosis. Moreover, these findings suggest that MK may be both a biomarker and therapeutic molecular target for malignant neoplasms.

In conclusion, serum MK levels in patients with HNSCC were associated with malignancy and chemosensitivity to ICT and were an independent prognostic factor. Because MK is a secreted soluble protein, it is detectable in peripheral blood examinations. Therefore, serum MK may be a useful, minimally invasive biomarker for detecting malignancy early, selecting appropriate patients for ICT, and predicting prognosis in HNSCC.

## Conflict of Interest

None declared.
